# The association between serum albumin and long length of stay of patients with acute heart failure: A retrospective study based on the MIMIC-IV database

**DOI:** 10.1371/journal.pone.0282289

**Published:** 2023-02-24

**Authors:** Tao Liu, Haochen Xuan, Lili Wang, Xiaoqun Li, Zhihao Lu, Zhaoxuan Tian, Junhong Chen, Chaofan Wang, Dongye Li, Tongda Xu

**Affiliations:** 1 Department of Cardiology, Jinshan Branch of Shanghai Sixth People’s Hospital, Jinshan, Shanghai, China; 2 Department of Cardiology, The Affiliated Hospital of Xuzhou Medical University, Xuzhou, Jiangsu, China; Azienda Ospedaliero Universitaria Careggi, ITALY

## Abstract

**Background:**

The purpose of this article is to assess the relationship between serum albumin level and long length of stay (LOS) of inpatients with acute heart failure (AHF) in the intensive care unit (ICU).

**Methods:**

We retrospectively analyzed data of 2280 patients with AHF from the medical information mart for intensive care IV (the MIMIC-IV) database. Multivariate logistic regression was performed to evaluate the association between serum albumin and long LOS, and the development of the predictive model was based on independent predictors of long LOS.

**Results:**

According to the statistical results, A negative linear relationship was presented between albumin and long LOS of AHF patients in the ICU (*P* for trend <0.001), and serum albumin could predict long LOS (AUC 0.649, 95%CI 0.616–0.683, *P* <0.001). Based on independent predictors, including respiratory failure (OR 1.672, 95%CI 1.289–2.169, *P*<0.001), WBC (OR 1.046, 95%CI 1.031–1.061, *P*<0.001), creatinine (OR 1.221, 95%CI 1.098–1.257, *P*<0.001), glucose (OR 1.010, 95%CI 1.007–1.012, *P*<0.001), lactic acid (OR 1.269, 95%CI 1.167–1.381, *P*<0.001), and albumin (OR 0.559, 95%CI 0.450–0.695, *P*<0.001), identified by multivariable logistic regression analysis, we developed the nomogram to predict the probability of long LOS of AHF patients in the ICU. The nomogram accurately predicted the probability of long LOS (AUC 0.740, 95%CI 0.712–0.768, *P*<0.001). The calibration suggested the predictive probability was highly consistent with the actual probability of long LOS. Decision curve analysis (DCA) also suggested that the nomogram was applicable in the clinic.

**Conclusion:**

Serum albumin level was negatively associated with LOS among AHF patients. The predictive model based on serum albumin has predictive value for evaluating the length of stay in AHF patients.

## Introduction

As a common and growing medical problem, heart failure (HF) is one of the most frequent causes of admission and hospital mortality [1]. The limitation of options for the management of patients with HF leads to a long length of stay (LOS). Especially, some patients with acute heart failure (AHF) in the intensive care unit (ICU) might have many underlying diseases, which might lead to long LOS. As a major problem of cardiovascular disease, AHF not only seriously affects the quality of life of patients, but also weighs their economic burden. In addition, whether longer LOS is a predictor of hospital readmission in patients with heart failure is controversial, but the strong association with the occurrence of in-hospital worsening heart failure (WHF) and early mortality of AHF patients was confirmed [2–4]. Therefore, we need to pay more attention to LOS.

As an inexpensive and powerful indicator, serum albumin is an essential protein in the human body produced by the liver and often used to evaluate the prognosis of a variety of diseases, such as HF, acute pulmonary embolism, diabetic nephropathy, and patients with severe illness in ICU [5–8]. Currently, serum albumin level has been added as one of the component parameters in the Acute Physiology and Chronic Health Evaluation (APACHE) III score [9]. Additionally, previous studies have shown that serum albumin was an independent indicator for the prognosis of patients with HF, and high albumin level was associated with the reduction of hospital mortality and rehospitalization [10–12]. Therefore, it is important to improve the albumin levels of these patients to reduce mortality, shorten the LOS and thus decrease the medical costs. Hypoalbuminemia often occurs in ICU patients [13], and it is closely related to the long LOS. However, there was no study on whether serum albumin level affects LOS in AHF patients in ICU.

Therefore, In the present study, we analyzed the association between serum albumin level and LOS of patients with AHF from the medical information mart for intensive care IV (the MIMIC-IV) database. what’s more, based on albumin, we developed a prediction model that can predict the length of hospital stay.

## Materials and methods

### Study population

**Fig 1:**
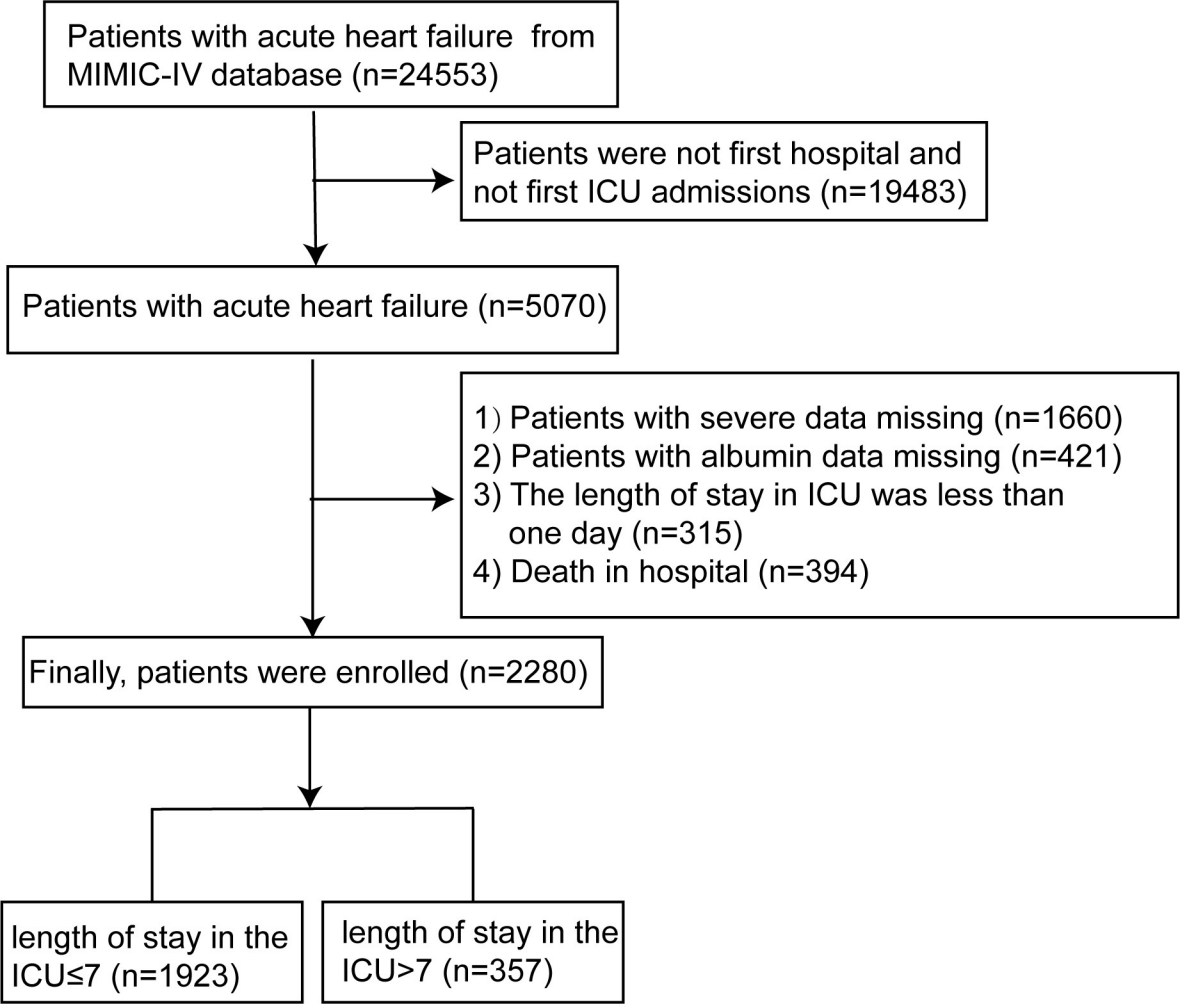
Flow chart of being included patients with acute heart failure. Abbreviations: MIMIC-IV, the medical information mart for intensive care IV; ICU, intensive care unit.

Two thousand, and two hundred and eighty AHF patients whose years more than 18 were enrolled from the MIMIC-IV database (AHF icd_code = 42821, 42823, 42831, 42833, 42841, 42843, I5021, I5023, I5031, I5033, I5041, I5043, I50811, I50813) in our study. The data provided by the above database was the original data. Thus, data used for the study was retrieved from MIMIC-IV by author Liu (ID: 9008147), who had completed the online training course of the National Institutes. The selection process of study participants is shown in **Fig 1**. This study was approved by the Institutional Review Boards of Beth Israel Deaconess Medical Center (Boston, MA) and the Massachusetts Institute of Technology (Cambridge, MA), and the requirement for patient informed consent was waived due to the study design.

### Variables

The exposure variable of the present study was serum albumin, and it was defined as the first serum albumin level at the time of admission to ICU. The outcome variable was the LOS of ICU, A "long length of stay" in the ICU was considered when more than 7 days. Categorical variables [including gender, ethnicity, insurance, comorbidities, and drugs use] and continuous variables were collected for all patients, including relevant demographic variables [including age, systolic blood pressure (SBP), diastolic blood pressure (DBP), and body mass index (BMI)] and blood biomarkers [white blood cell (WBC), creatinine, bun, calcium, sodium, potassium, lactic acid, glucose, hematocrit, platelet, and hemoglobin]. For patients who had been admitted to the ICU more than once, the clinical data of their first admission to the ICU was collected.

### Statistical analysis

Continuous variables were represented by mean ± standard deviation or median (25th quartile, 75th quartile) in the table. If the test of normality and the homogeneity test of variance is performed, the T-test is appropriate, otherwise, the Kruskal Wallis test is appropriate. Categorical variables are presented as a percentage in the tables and compared using the *X*^*2*^ test. Multivariate logistic regression with a significant difference of 0.05 was used to build the final models, and nomogram was developed by results of multivariate logistic regression. Discriminative capacity, calibration ability, and clinical effectiveness were used to evaluate nomogram. The concordance index (C-index) was measured to quantify the discrimination capacity of the nomogram, and C-index is equal to the area under the receiver operating characteristics curve (AUC-ROC) in logistic regression analysis. A calibration plot was drawn to evaluate the prediction accuracy of the nomogram. Decision curve analysis (DCA) was performed to evaluate Clinical effectiveness. All statistical analyses were performed using the software Stata15 (Version 15.0, https://www.stata.com/) and package *R* (http://www.Rproject.org). All *P*-value <0.05 were significant.

## Results

### Baseline characteristics

The mean age of 2280 patients were 69.88 ± 13.72, and men made up 56.80% of the total. Compared with the group with ICU stay less than 7 days, patients of the group with ICU stay longer than 7 days were more likely to have respiratory failure, sepsis, and high use ratio of nitroglycerin, and increased levels of blood glucose, creatinine, Bun, WBC, calcium, lactic acid value, and decreased level of albumin value (all P<0.05). Except for the above variables (*P* < 0.05), all variables were not significant (*P* > 0.05) (**Table 1**).

**Table 1:** Baseline characteristics.

Variables	Total (n = 2280)	LOS≤7 (n = 1923)	LOS>7 (n = 357)	P-value
Age, years	69.88 ± 13.72	69.99 ± 13.75	69.26 ± 13.57	0.297
Sex, n (%)				0.543
Male	1295 (56.80)	1087 (56.53)	208 (58.26)	
Female	985 (43.20)	836 (43.47)	149 (41.74)	
Ethnicity, n (%)				0.082
Whites	1547 (67.85)	1305 (67.86)	242 (67.79)	
Black	196 (8.60)	175 (9.10)	21 (5.88)	
Other	537 (23.55)	443 (23.04)	94 (26.33)	
Insurance, n (%)				0.509
Medicare	1268 (55.61)	1073 (55.80)	195 (54.62)	
Medicaid	119 (5.22)	104 (5.41)	15 (4.20)	
Other	893 (39.17)	746 (38.79)	147 (41.18)	
BMI, kg/m^2^	29.12 ± 6.47	29.13 ± 6.44	29.08 ± 6.67	0.654
SBP, mmHg	115.33 ± 20.77	115.62 ± 20.76	113.78 ± 20.77	0.093
DBP, mmHg	57.84 ± 16.53	57.76 ± 16.63	58.28 ± 15.95	0.729
Respiratory failure, n (%)	787 (34.52)	606 (31.51)	181 (50.70)	<0.001
Sepsis, n (%)	148 (6.49)	110 (5.72)	38 (10.64)	<0.001
COPD, n (%)	253 (11.10)	213 (11.08)	40 (11.20)	0.944
Malignancy, n (%)	11 (0.48)	8 (0.42)	3 (0.84)	0.288
AF, n (%)	1226 (53.77)	1025 (53.30)	201 (56.30)	0.296
Liver cirrhosis, n (%)	44 (1.93)	39 (2.03)	5 (1.40)	0.429
AMI, n (%)	320 (14.04)	274 (14.25)	46 (12.89)	0.496
CKD, n (%)	893 (39.17)	760 (39.52)	133 (37.25)	0.42
Diabetes, n (%)	435 (19.08)	372 (19.34)	63 (17.65)	0.453
Hypertension, n (%)	100 (4.39)	85 (4.42)	15 (4.20)	0.853
Diuretics, n (%)	2203 (96.62)	1858 (96.62)	345 (96.64)	0.986
β blocker, n (%)	1968 (86.32)	1665 (86.58)	303 (84.87)	0.388
Nitroglycerin, n (%)	1306 (57.28)	1131 (58.81)	175 (49.02)	<0.001
WBC, ×10^9^/L	13.36 ± 8.28	12.59 ± 6.65	17.53 ± 13.41	<0.001
Creatinine, mg/dL	1.58 ± 1.24	1.53 ± 1.16	1.89 ± 1.59	0.011
Bun, mg/dl	33.56 ± 22.22	32.96 ± 21.65	36.76 ± 24.82	0.016
Calcium, mg/dl	8.67 ± 0.75	8.70 ± 0.74	8.54 ± 0.78	<0.001
Sodium, mmol/L	138.39 ± 4.83	138.36 ± 4.66	138.55 ± 5.68	0.75
Potassium, mmol/L	4.18 ± 0.60	4.19 ± 0.61	4.13 ± 0.56	0.151
Lactic acid, mmol/L	1.98 ± 1.28	1.90 ± 1.15	2.39 ± 1.78	0.004
Glucose, mg/dL	133.15 ± 52.33	129.21 ± 48.93	154.37 ± 63.78	<0.001
Hematocrit, %	31.94 ± 6.42	32.00 ± 6.39	31.60 ± 6.56	0.179
Platelet, ×10^9^/L	226.46 ± 112.06	226.60 ± 109.63	225.68 ± 124.50	0.356
Hemoglobin, g/dl	10.33 ± 2.14	10.36 ± 2.14	10.15 ± 2.14	0.062
Albumin, g/dL	3.46 ± 0.63	3.50 ± 0.61	3.23 ± 0.69	<0.001

Abbreviations: BMI, body mass index; SBP, systolic blood pressure; DBP, diastolic blood pressure; COPD, chronic obstructive pulmonary disease; AF, atrial fibrillation; AMI, acute myocardial infarction; CKD, Chronic kidney disease; WBC, white blood cell. Data are present as MD±SD or N (%).

### Analysis of association between serum albumin and long length of stay in the intensive care unit

**Fig 2:**
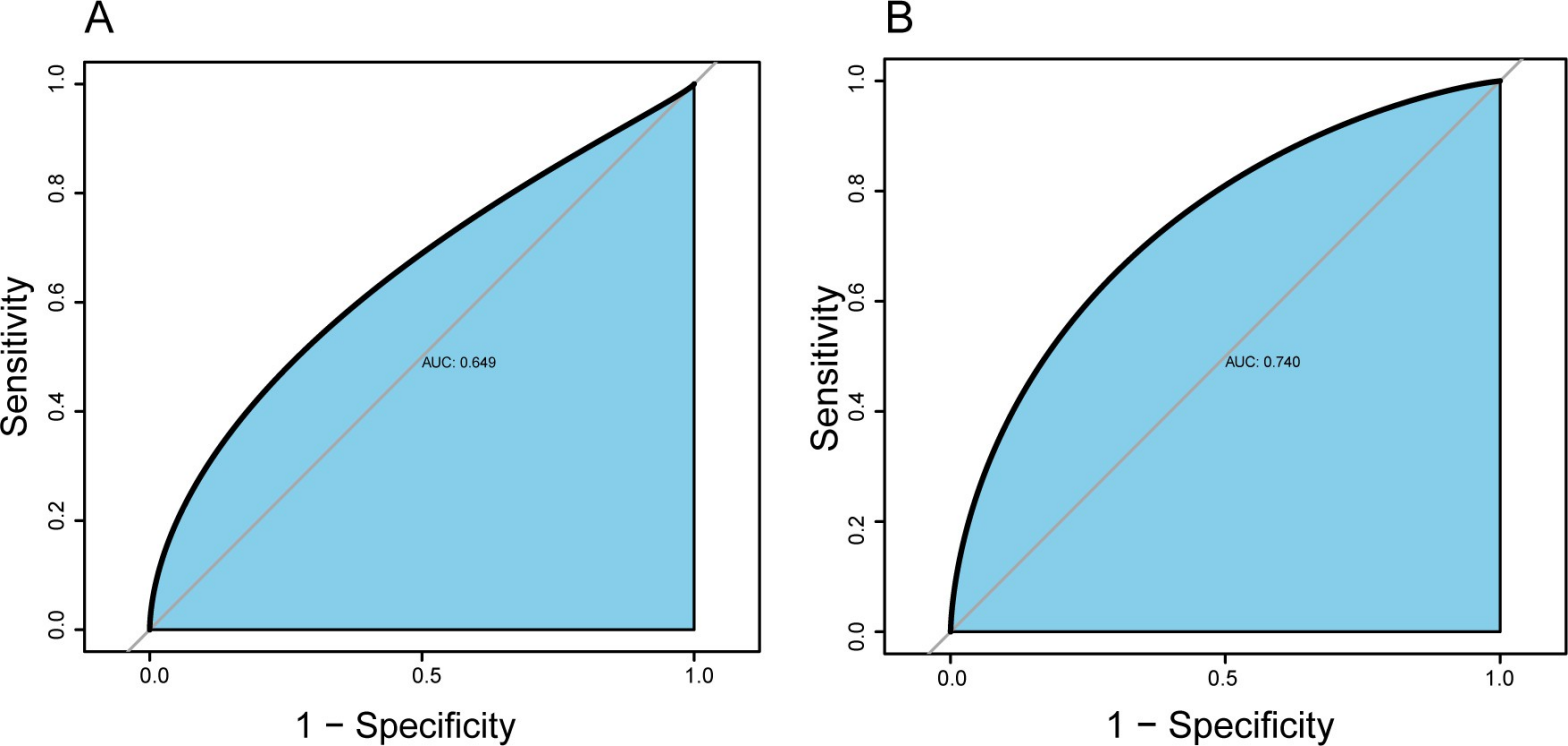
The ROC curve for serum albumin to predict long length of stay of AHF patients in the ICU (A). The ROC curve for the predictive model to predict long length of stay of AHF patients in the ICU (B). Abbreviations: ROC, receiver operating characteristic; AHF, acute heart failure; ICU, intensive care unit.

We further analyzed the relationship between serum albumin and long LOS in the ICU. In the final adjusted model, serum albumin could be a protective factor for LOS shortening of patients with AHF (OR: 0.559; 95%CI: 0.450–0.695; *P* <0.001). the OR (95% CI) for long LOS across the albumin quartile were 0.558 (0.402–0.774), 0.532 (0.370–0.765), and 0.455 (0.312–0.662), compared with the first quartile of albumin (*P* for trend <0.001). Both unadjusted and adjusted only age, gender, and ethnicity models showed statistically similar significance to the above (**Table 2**). **Fig 2A** showed that serum albumin could predict a long length of stay in the ICU (AUC 0.649, 95%CI 0.616–0.683, *P* <0.001).

**Table 2:** Association of serum albumin with length of stay in the intensive care unit.

Variables	Model 1	Model 2	Model 3
	OR (95%CI) *P*-value	OR (95%CI) *P*-value	OR (95%CI) *P*-value
Albumin	0.432 (0.360, 0.521) <0.001	0.433 (0.359, 0.521) <0.001	0.559 (0.450, 0.695) <0.001
Quartile of albumin			
Q1	1.0	1.0	1.0
Q2	0.453 (0.337, 0.609) <0.001	0.455 (0.339, 0.612) <0.001	0.558 (0.402, 0.774) <0.001
Q3	0.398 (0.289, 0.548) <0.001	0.340 (0.290, 0.550) <0.001	0.532 (0.370, 0.765) <0.001
Q4	0.299 (0.217, 0.412) <0.001	0.298 (0.216, 0.411) <0.001	0.455 (0.312, 0.662) <0.001
*P* for trend	<0.001	<0.001	<0.001

Quartiles of albumin: Q1, 1.6–3.0g/dL; Q2, 3.1–3.5g/dL; Q3, 3.6–3.9g/dL; Q4, 4.0–5.1g/dL. Model 1: None was adjusted. Model 2: Age, gender and ethnicity were adjusted. Model 3: white blood cell, hemoglobin, glucose, creatinine, bun, calcium, lactic acid, respiratory failure, sepsis and nitroglycerin were adjusted. Abbreviations: CI, confidence interval.

### Development and evaluation of the predictive model

The predictors with *P-*value <0.05, including respiratory failure (OR 2.320, 95%CI 1.851–2.909, *P*<0.001), sepsis (OR 2.195, 95%CI 1.507–3.198, *P*<0.001), nitroglycerin (OR 0.651, 95%CI 0.521–0.814, *P*<0.001), WBC (OR 1.061, 95%CI 1.047–1.075, *P*<0.001), hemoglobin (OR 0.944, 95%CI 0.895–0.996, *P =* 0.036), creatinine (OR 1.219, 95%CI 1.130–1.315, *P*<0.001), Bun (OR 1.007, 95%CI 1.002–1.012, *P* = 0.003), calcium (OR 0.719, 95%CI 0.617–0.837, *P*<0.001), glucose (OR 1.010, 95%CI 1.008–1.012, *P*<0.001), lactic acid (OR 1.275, 95%CI 1.182–1.375, *P*<0.001), and albumin (OR 0.433, 95%CI 0.360–0.521, *P*<0.001), were selected by the univariate analysis results (**Table 3**). Finally, the independent influencing factors for long LOS for AHF patients in the ICU were selected by multivariate regression analysis, including respiratory failure (OR 1.672, 95%CI 1.289–2.169, *P*<0.001), WBC (OR 1.046, 95%CI 1.031–1.061, *P*<0.001), creatinine (OR 1.221, 95%CI 1.098–1.257, *P*<0.001), glucose (OR 1.010, 95%CI 1.007–1.012, *P*<0.001), lactic acid (OR 1.269, 95%CI 1.167–1.381, *P*<0.001), and albumin (OR 0.559, 95%CI 0.450–0.695, *P*<0.001) (**Table 4**).

**Table 3:** Univariate logistic regression analysis for predicting long length of stay of acute heart failure in the intensive care unit.

Variables	Unadjusted logistic regression	
	OR	*P*-value
Age, years	0.996 (0.988,1.004)	0.275
Sex, n (%)		0.732
male	1.0	
female	0.961 (0.767,1.205)	
Ethnicity, n (%)		0.313
whites	1.0	
black	0.647 (0.403,1.038)	0.071
other	1.144 (0.881,1.486)	0.312
Insurance, n (%)		0.494
medicare	1.0	
medicaid	0.794 (0.452,1.393)	
other	1.084 (0.858,1.370)	
BMI, kg/m^2^	0.997 (0.980,1.014)	0.712
SBP, mmHg	0.996 (0.991,1.002)	0.179
DBP, mmHg	1.002 (0.995,1.008)	0.652
COPD, n (%)		0.88
No	1.0	
Yes	0.973 (0.680,1.391)	
Malignancy, n (%)		0.323
No	1.0	
Yes	1.956 (0.517,7.408)	
AF, n (%)		0.354
No	1.0	
Yes	1.112 (0.888,1.392)	
Liver cirrhosis, n (%)		0.649
No	1.0	
Yes	0.817 (0.343,1.948)	
AMI, n (%)		0.446
No	1.0	
Yes	0.879 (0.631,1.224)	
CKD, n (%)		0.406
No	1.0	
Yes	0.907 (0.720,1.142)	
Diabetes, n (%)		0.542
No	1.0	
Yes	0.914 (0.684,1.221)	
Hypertension, n (%)		0.751
No	1.0	
Yes	0.913 (0.521,1.600)	
Respiratory failure, n (%)		<0.001
No	1.0	
Yes	2.320 (1.851,2.909)	
Sepsis, n (%)		<0.001
No	1.0	
Yes	2.195 (1.507,3.198)	
Diuretics, n (%)		0.857
No	1.0	
Yes	0.946 (0.515,1.735)	
β blocker, n (%)		0.206
No	1.0	
Yes	0.819 (0.601, 1.116)	
Nitroglycerin, n (%)		<0.001
No	1.0	
Yes	0.651 (0.521,0.814)	
WBC, ×10^9^/L	1.061 (1.047,1.075)	<0.001
Hemoglobin, g/dl	0.944 (0.895,0.996)	0.036
Platelet, ×10^9^/L	1.000 (0.999,1.001)	0.973
Creatinine, mg/dL	1.219 (1.130,1.315)	<0.001
Bun, mg/dl	1.007 (1.002,1.012)	0.003
Calcium, mg/dl	0.719 (0.617,0.837)	<0.001
Glucose, mg/dL	1.010 (1.008,1.012)	<0.001
Sodium, mmol/L	1.014 (0.991,1.038)	0.244
Potassium, mmol/L	0.828 (0.681,1.006)	0.057
Lactic acid, mmol/L	1.275 (1.182,1.375)	<0.001
Hematocrit, %	0.986 (0.969,1.004)	0.127
Albumin, g/dL	0.433 (0.360,0.521)	<0.001

Abbreviations: BMI, body mass index; SBP, systolic blood pressure; DBP, diastolic blood pressure; COPD, chronic obstructive pulmonary disease; AF, atrial fibrillation; AMI, acute myocardial infarction; CKD, Chronic kidney disease; WBC, white blood cell.

**Table 4:** Multivariate logistic regression analysis for predicting long length of stay of acute heart failure in the intensive care unit.

Variables	Adjusted logistic regression	
	OR 95%CI	*P*-value
Respiratory failure, n (%)	1.672 (1.289,2.169)	<0.001
WBC, ×10^9^/L	1.046 (1.031,1.061)	<0.001
Creatinine, mg/dL	1.221 (1.098,1.257)	<0.001
Glucose, mg/dL	1.010 (1.007,1.012)	<0.001
Lactic acid, mmol/L	1.269 (1.167,1.381)	<0.001
Albumin, g/dL	0.559 (0.450,0.695)	<0.001

Abbreviations: WBC, white blood cell.

**Fig 3:**
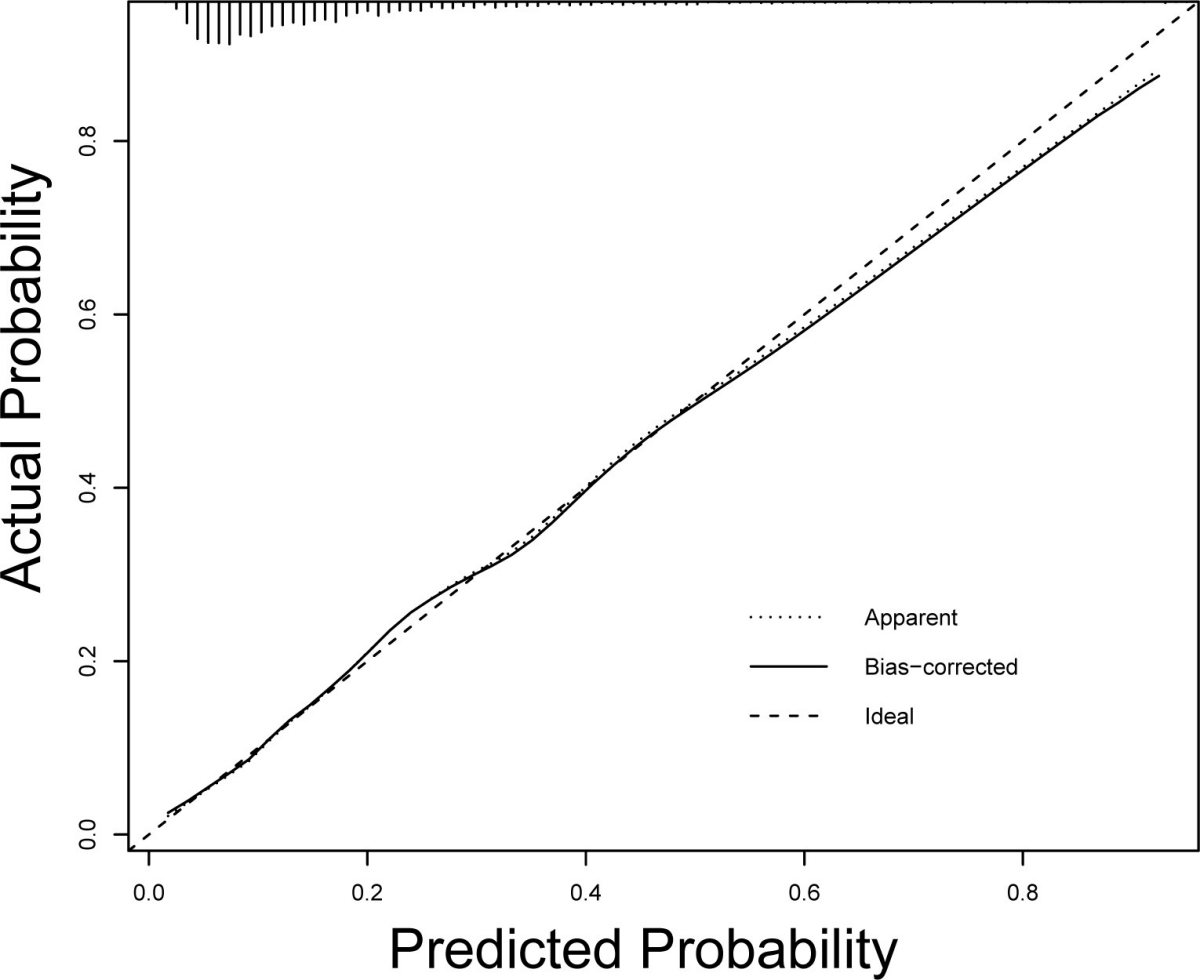
The calibration plot for the nomogram to predict long length of stay. The predicted probability of long length of stay is plotted on the x-axis and the actual probability is plotted on the y-axis.

**Fig 4:**
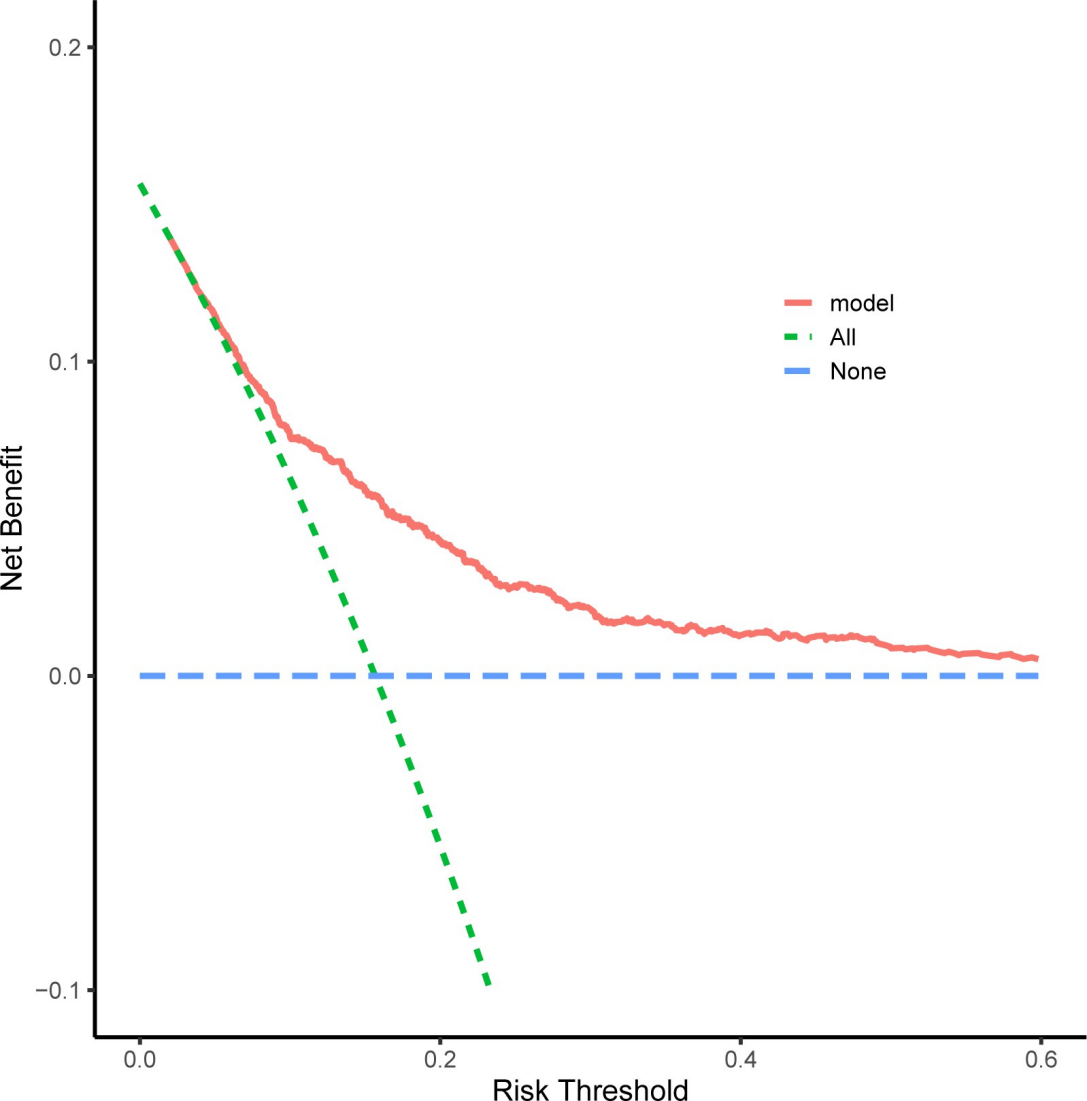
The decision curve analysis (DCA) for the nomogram to predict long length of stay. The blue dotted line represents the intervention-none and the net benefit with zero, the green dotted line shows intervention-all-patients. The upper part of the green dotted line represents positive income, meanwhile, the lower part represents negative income. The red line represents a threshold of the model.

**Fig 5:**
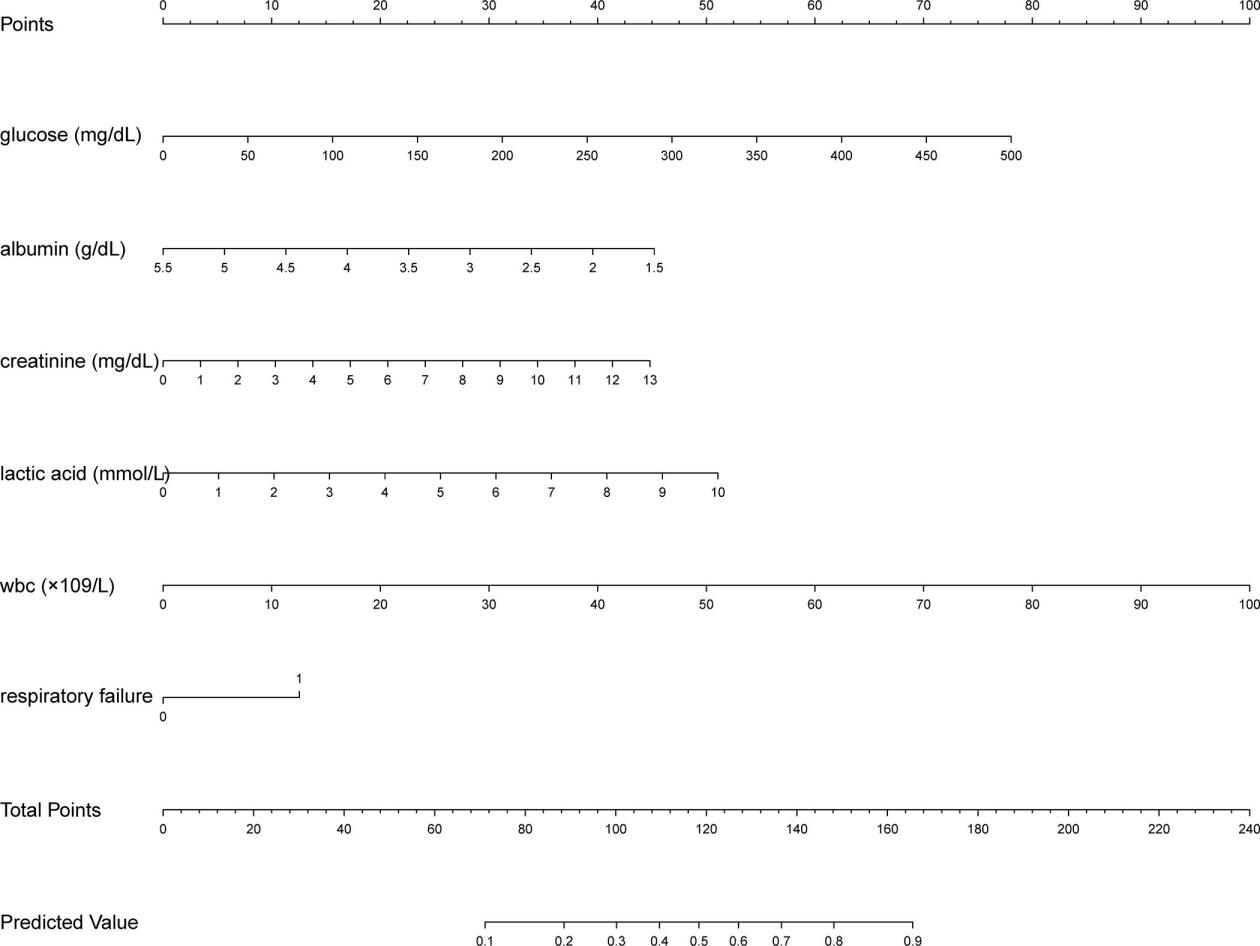
Nomogram for predicting the possibility of long length of stay of AHF patients in the ICU. The score of each variable is calculated by drawing a vertical line between each variable axis and the top line of the nomogram. Then, we can add the scores of each variable and find the corresponding score on the total scoreline. Finally, we can draw a vertical line from the total scoreline to the bottom predicted probability scale to obtain the individual probability of long length of stay of AHF patients in the ICU. Abbreviations: AHF, acute heart failure; ICU, intensive care unit.

Based on the above independent influencing factors, we developed a predictive model. The AUC of the predictive model for predicting the long LOS of AHF patients was 0.740 (95%CI 0.712–0.768, *P*<0.001), which suggested that the predictive model has a better discriminative capacity (**Fig 2B**). The predictive model has high consistency for predicted and actual probability for predicting long LOS (**Fig 3**). When the threshold probability is greater than 5%, the predictive model has a high net benefit in assessing the prognosis of AHF patients, which suggests that the predictive model was clinically useful (**Fig 4**). Finally, the predictive model was visualized by nomogram (**Fig 5**).

## Discussion

The present study uncovered that serum albumin was a linearly negative correlation to LOS of patients with AHF. What’s more, the nomogram developed based on serum albumin level can predict the length of hospital stay for AHF patients.

The nutritional status of AHF patients in ICU significantly affects their prognosis and LOS. Despite albumin playing an important role in their nutritional status, the information from previous studies linking serum albumin and LOS is limited. Recently, Yanni and their colleagues studied the relationship between albumin and LOS of critically ill children. Although LOS (mean +SD) in the hypoalbuminemia group was higher than that in the normal group, their data revealed statistically that increased serum albumin levels did not shorten LOS or decrease clinical mortality [14]. However, a meta-analysis study compositing of 291433 participants conducted by Vincent et al, showed each 10 g/L declines in serum albumin concentration significantly raised prolonged intensive care unit stay by 28% [15]. In the present study, the participants were AHF patients in ICU from the MIMIC-IV database from 2008–2019, and with the adjustments of the variables, the higher serum albumin level should be considered as beneficial for LOS shortening.

Serum albumin not only plays an important role in their nutritional statue, but also maintains the colloidal osmotic pressure. For AHF patients, hypoalbuminemia will reduce the patient’s blood volume, which will lead to insufficient perfusion of important organs, thereby aggravating the patient’s clinical symptoms. Previous studies have shown that hypoalbuminemia significantly affected prognosis and LOS of various diseases, such as patients with severe sepsis, acute decompensated chronic obstructive pulmonary disease, and requiring surgery [16–18], but the relationship between albumin and length of stay in AHF patients has not been studied. In the present study, negative relationships between serum albumin and LOS in patients with AHF were found. Serum albumin could maintain the colloidal osmotic pressure, the proper serum albumin is beneficial to the recovery of AHF patients. Currently, several studies had suggested that serum albumin level <3.5g/dL was an independent risk factor for prolonged LOS [16, 17, 19], Clinicians are more inclined to treat moderate and severe hypoalbuminemia, but albumin levels are divided into normal albumin levels (≥3.5g/dl), mild hypoalbuminemia (3.0g/dl-3.5g/dl), moderate hypoalbuminemia (2.5g/dl-3.0g/dl) and severe hypoalbuminemia (<2.5g/dl). Based on current research results, the treatment of mild albuminemia is also vital to shorten the LOS.

In addition, the results of the present study also found respiratory failure, WBC, creatinine, glucose, and lactic acid were independent risk factors for long LOS among AHF patients. The related reasons are as follows. For AHF patients, respiratory failure increases hypoxia symptoms and internal environmental disturbances, which leads to a long LOS. Previous studies have shown that patients with respiratory failure have low albumin levels due to severe metabolic depletion [20]. Severe malnutrition could also lead to long LOS. The meta-analysis of 5 pooled clinical trials by Miller et al. found that respiratory failure was associated with poor prognosis among AHF patients [21]. In addition, the LOS of older adults with acute respiratory failure will be prolonged [22]. Consistent with the results of this study, respiratory failure is a strong independent risk factor for long LOS of AHF patients. In addition, Heim et al. found that WBC could predict hospital LOS and disease severity in patients with odontogenic abscess [23]. Meanwhile, infection is an important cause of the occurrence of AHF. Chien et al. found that in the Asian population of patients with acute heart failure with preserved ejection fraction (HFpEF), patients with hypoalbuminemia often have systemic inflammation, and malnutrition plays a key role in the prognosis of AHF patients [24]. Similarly, WBC, an indicator of inflammation, was found to be an independent predictor of long LOS in AHF patients in this study. Moreover, the abnormal renal function reduces the effect of diuretics in improving the symptoms of AHF patients, and hypoalbuminemia can further cause water and sodium retention. these would aggravate the symptoms of AHF patients and prolong the LOS. Cardiovascular diseases could induce or aggravate renal dysfunctions, in this way further deteriorating cardiac function and creating a vicious circle [25]. In AHF patients, hyperglycemia may be the response to the danger and is a reflection of an activated sympathetic nervous system. Hyperglycemia in hospitalized patients may be caused not only by the poor glycemic control in diabetes but also by a transient stress response to current disease states [26]. Previous studies have confirmed hyperglycemia at admission is independently associated with hospitalization and short-term mortality in AHF patients and is an independent predictor of 1-year mortality in non-diabetic (DM) patients with AHF [27, 28]. Furthermore, higher preoperative blood glucose level was significantly related to a prolonged LOS for patients undergoing appendectomy or laparoscopic cholecystectomy [29]. Lactic acid, as a mesostate of blood glucose, is produced mainly by glycolysis due to stress or hypoxia. In the setting of AHF, peripheral hypoperfusion and low cardiac output can alter the lactate homeostasis, and promote lactate accumulation [30]. Lactate is a reliable predictor of increased LOS in surgical patients [31, 32]. Multiple studies have confirmed that elevated lactate levels are useful for identifying high-risk patients and for predicting worse outcomes and the high risk of mortality in patients with AHF [33, 34]. Similarly, we found that lactate level was closely related to increased LOS in the present study.

Finally, the present study found that high serum albumin level was beneficial to LOS shortening of AHF patients, and the result could guide clinicians to reduce LOS of AHF patients by changing albumin levels. In addition, the predictive model we developed could identify high-risk patients. However, our study also has some limitations. Firstly, the maximum serum albumin level included in our study was 5.1 g/dl. When the albumin level is greater than 5.1 g/dl, whether the relationship between serum albumin and LOS is still negative has not been studied. Secondly, we only focus on the development of the model without external verification. Additional experiments may be required for external verification. Finally, we might not adjust other potential confounding factors.

## Conclusions

Serum albumin level was negatively associated with long LOS among AHF patients. The predictive model based on serum albumin has predictive value for evaluating the long LOS in AHF patients.
